# Voltammetric Determination of Ferulic Acid Using Polypyrrole-Multiwalled Carbon Nanotubes Modified Electrode with Sample Application

**DOI:** 10.3390/nano5041704

**Published:** 2015-10-16

**Authors:** Refat Abdel-Hamid, Emad F. Newair

**Affiliations:** Unit of Electrochemistry Applications (UEA), Chemistry Department, Faculty of Science, Sohag University, Sohag 82524, Egypt; E-Mail: refat.abdelhamid@science.sohag.edu.eg

**Keywords:** voltammetry, polypyrrole-multiwalled carbon nanotubes, ferulic acid, modified electrode

## Abstract

A polypyrrole-multiwalled carbon nanotubes modified glassy carbon electrode-based sensor was devised for determination of ferulic acid (FA). The fabricated sensor was prepared electrochemically using cyclic voltammetry (CV) and characterized using CV and scanning electron microscope (SEM). The electrode shows an excellent electrochemical catalytic activity towards FA oxidation. Under optimal conditions, the anodic peak current correlates linearly to the FA concentration throughout the range of 3.32 × 10^−6^ to 2.59 × 10^−5^ M with a detection limit of 1.17 × 10^−6^ M (*S/N* = 3). The prepared sensor is highly selective towards ferulic acid without the interference of ascorbic acid. The sensor applicability was tested for total content determination of FA in a commercial popcorn sample and showed a robust functionality.

## 1. Introduction

Ferulic acid (FA) is a superior antioxidant among many ubiquitous polyphenolic acids in the plant kingdom. Ferulic acid plays a major role in protecting cell constituents; therefore, its longer circulation in the blood stream is of great interest. Moreover, FA is actively used as an anti-inflammatory, anti-aging, and antithrombotic drug [1]. Additionally, it alleviates oxidative stress as well as decreases glucose levels in diabetic rats [2]. For all aforementioned facts, it is of significant importance to develop a simple, effective, fast, and low-cost method for the determination of ferulic acid.

Several analytical methodologies are available for the determination of ferulic acid concentration, *i.e.*, high-performance liquid chromatography (HPLC) [3] and spectrophotometry [4]. Such instrumental methods depend on multistep sample cleanup procedures, which are time-consuming, pricey, and of complex setup. However, the electroanalytical approach has become an alternative method for analysis of ferulic acid due to many reasons, such as high selectivity, quick response, low cost, possible miniaturization, and ease of operation. Recently, several types of sensors were developed for the determination of FA, such as the l-cysteine self-assembled monolayers (SAM) modified gold electrode [5], carbon paste electrode [6,7], multiwalled carbon nanotube-modified glassy carbon electrode (MWCNTs/GCE) [8], multiwalled carbon nanotubes decorated with MnO_2_ nanoparticle-modified GCE [9], and reduced graphene oxide/GCE [10]. Moreover, a rapid electrochemical detection of ferulic acid using a graphene sensor was also introduced [11]. For the direct quantitative determination of FA, a clever and more sensitive sensor based on a poly(diallyldimethylammoniumchloride) functionalized graphene-modified GCE was introduced [1]. Nevertheless, it remains a fruitful challenge to build an electrochemical sensor based on novel nano-materials for performing effortless, sensitive, and fast detection of ferulic acid.

Composite materials based on conjugation of conducting polymers and CNTs were shown to possess properties of individual components with a harmonic effect. Numerous efforts were committed to design and prepare new polymer-CNT composites, which exhibit new features in specific applications. Peng *et al.* studied the fabrication of composite films of CNTs with polyaniline (PANI), polypyrrole (PPy) or poly[3,4-ethylenedioxythiophene] (PEDOT) via electrochemical co-deposition methods, using a solution of acid-treated CNTs and the corresponding monomer. The electrochemically synthesized composite films have a porous structure at the micro- and nano-meter scales in common. In addition, they have better mechanical integrity, higher conductivity, and greater stability compared to pure conducting polymers [12]. It was shown that a polypyrrole carbon nanotube (PPy-CNTs) composite exhibits very different electronic properties compared to the sole composites of PPy and CNTs. Furthermore, composite films of CNTs with polypyrrole are well suited for gas sensor applications [13].

Throughout our experiment, pyrrole was selected as a starting monomer due to low cost and ease of electrochemical polymerization. The synthesized polypyrrole (PPy) has a substantial interest for its promising applications in sensing. Our choice of the PPy is based on good electronic and mechanical properties as well as high structural stability [14,15,16,17]. This research is a continuation of our previous work on the electrochemical study of antioxidants [18,19,20,21,22,23]. The aim is to establish a PPy-CNTs/GCE-based electrochemical sensor for the determination of FA. The composite electrode under study was prepared electrochemically and characterized using cyclic voltammetry (CV) and scanning electron microscope (SEM). Adsorptive square wave voltammetry is applied to validate the applicability of the sensor for the determination of total phenolic content and concentrations of FA in commercial popcorn.

## 2. Results and Discussion

### 2.1. Characterization of the Modified Electrode (PPy-MWCNTs/GCE)

**Figure 1:**
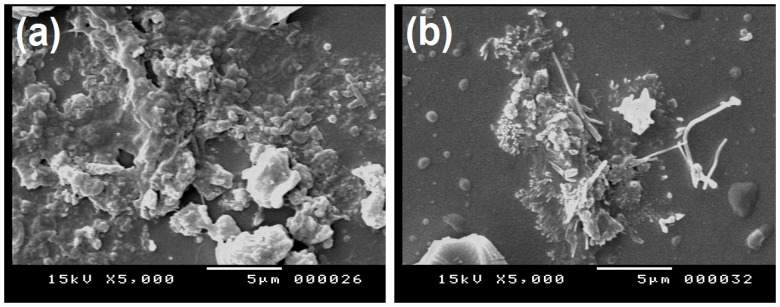
Scanning electron microscope (SEM) images of (**a**) polypyrrole (PPy) and (**b**) polypyrrole-multiwalled carbon nanotubes (PPy-MWCNTs) composite at indium tin oxide (ITO) electrode.

Figure 1 shows the SEM micrographs of pure PPy and PPy-MWCNTs composite thin films on an indium tin oxide (ITO) rectangular coated glass slide with a surface resistivity of 8–12 Ω/sq. The composite thin films are deposited on the ITO with the same electropolymerization conditions presented at the GCE experimental section. PPy film features irregular spheroid, grain-like structures and porous morphology (Figure 1a) compared to PPy-MWCNTs. The surface micrograph of the PPy-MWCNTs composite film has a uniform coating on the surface of PPy-conducting polymer (Figure 1b), which confirms the formation of PPy-MWCNTs composite.

**Figure 2:**
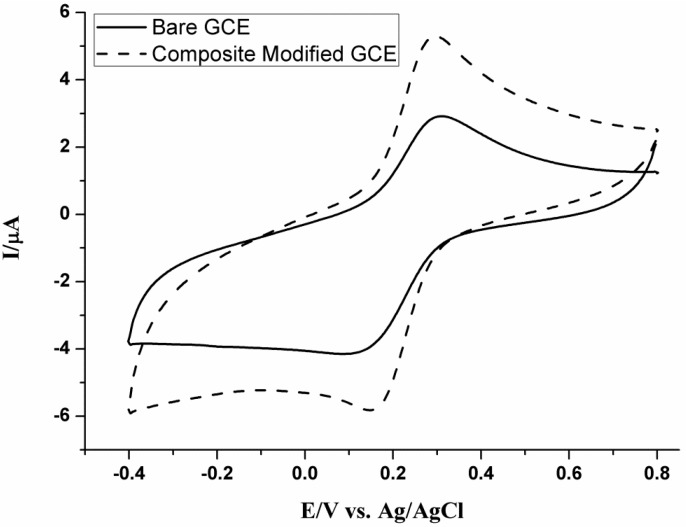
Cyclic voltammograms of 1.0 mM K_3_Fe(CN)_6_ in 0.2 M KCl on bare GCE and PPy-MWCNTs/GCE at a scan rate of 20 mV s^−1^.

Electrochemical properties of the bare GC and PPy-MWCNTs/GC electrodes were studied for 1.0 mM K_3_Fe(CN)_6_ solution, in 0.2 M KCl, using the cyclic voltammetric method at a scan rate of 20 mV/s. Cyclic voltammograms were performed and the results were depicted in Figure 2. K_3_Fe(CN)_6_ shows a pair of well-defined redox peaks with an enhanced peak current (5.29 μA) at PPy-MWCNTs/GCE compared to the bare GCE (2.91 μA), which enhanced the peak current by 1.82-folds. This indicates that PPy-MWCNTs film increases the active surface area of the electrode. Moreover, peak current enhancement accompanies the peak potential positive shift with peak potential separation, Δ*E*_pa_, of 10 mV. The preceding results confirm the abnormal electrocatalytic activity of the PPy-MWCNTs composite film. Our introduced modification increases the surface roughness and the effective surface area compared to the bare GCE. Roughness of the electrode surface of the PPy-MWCNTs/GC electrode is calculated by using the cyclic voltammetric Randles-Sevcik Equation (1):

(1) *i*_p_ = 2.69 × 10^5^*n*^3/2^*AD*^1/2^*Cv*^1/2^

where *i*_p_ is the peak current (A), *n* is the number of electrons, *A* is the active surface area (cm^2^), *D* is the diffusion coefficient (cm^2^/s), *C* is the concentration (mol/cm^3^), and *v* is the scan rate (V/s). For determining the ratio of the PPy-MWCNTs/GCE to the bare GCE, *i.e.*, the apparent surface active area, we need the data of the K_4_Fe(CN)_6_ cyclic voltammograms (Figure 2). The apparent surface active area ratio is found to be 1.31, which is similar to the results of Babaei *et al.* [24].

**Figure 3:**
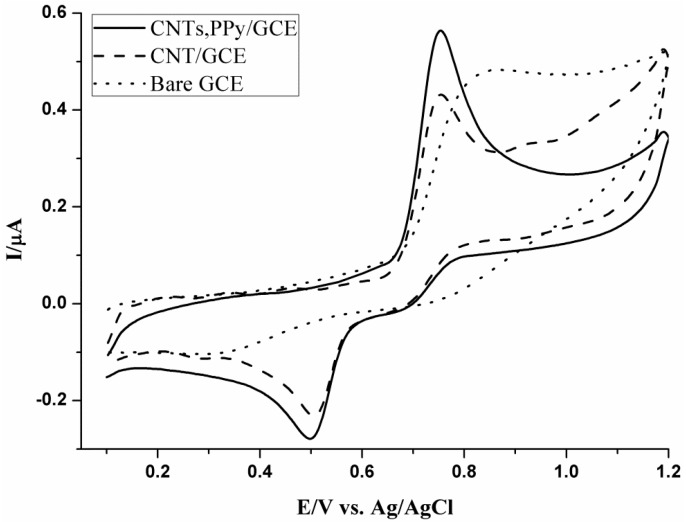
Cyclic voltammograms of 3.22 × 10^−5^ M FA on bare GCE, MWCNTs/GCE, and PPy-MWCNTs/GCE in 0.2 M H_3_PO_4_ (pH 2.54) at a scan rate of 20 mV/s.

Electrochemical properties of the bare GC and PPy-MWCNTs/GC electrodes are also studied using ferulic acid. Cyclic voltammetric behavior of 3.22 × 10^−5^ M FA on bare GC and the MWCNTs/GC electrodes was performed in 0.2 M H_3_PO_4_ (pH 2.54) at a scan rate of 20 mV s^−1^ and recorded in Figure 3. A broad oxidation and reduction waves at peak potentials *E*_pa_ of 850 mV and *E*_pc_ of 300 mV, respectively, are observed at the bare GCE, while at MWCNTs/GCE and PPy-MWCNTs/GCE, sharp waves are observed at 754 mV. The peak potential separation at bare GCE (Δ*E*_p_ = 550 mV) is higher than that at the modified one, which is (Δ*E*_p_ = 254 mV). This reveals that catalytic oxidation of the phenol groups of ferulic acid takes place on the modified electrodes at a less positive potential than that in the case of the bare electrode. As a result, an over-potential decrease by 96 mV and improvement of the electrochemical reversibility are obtained.

### 2.2. Square Wave Voltammetry of Ferulic Acid and Optimal Conditions of Accumulation

Voltammtric determination of ferulic acid was carried out, making use of the square wave adsorptive stripping method (SWAdsSV) on the modified glassy carbon electrode, PPy-MWCNTs/GCE. The SWAdsSV is an effective and rapid electroanalytical technique with advantages, including good discrimination against background currents and low detection limits. For the electrochemical determination of FA, optimal conditions such as accumulation potential, accumulation time, pH, and square frequency for FA are studied. Square wave voltammograms (SWVs) for 3.22 × 10^−5^ M ferulic acid on the modified electrode, PPy-MWCNTs/GCE, were recorded in 0.2 M H_3_PO_4_ (pH 2.54).

#### 2.2.1. Effect of Accumulation Potential

**Figure 4:**
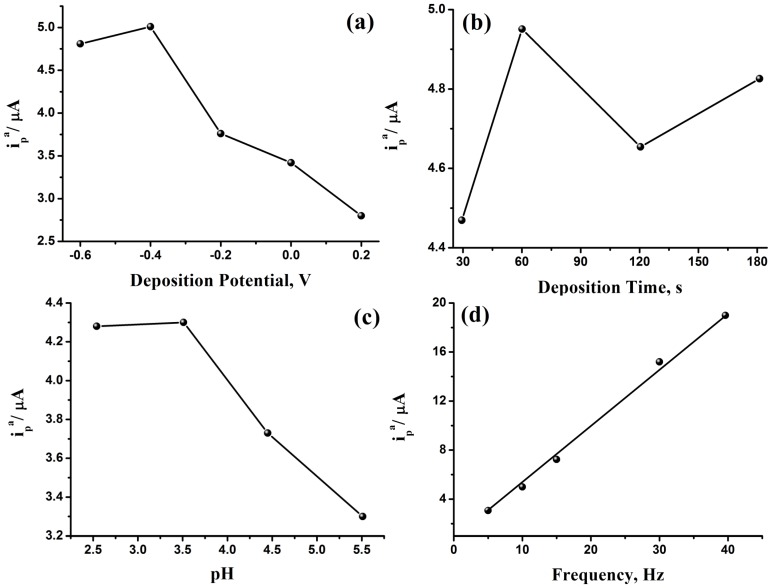
Effect of accumulation potential (**a**), accumulation time (**b**), and pH (**c**) on the anodic peak current of 3.22 × 10^−5^ M FA on PPy-MWCNTs/GCE in 0.2 M H_3_PO_4_ (pH 2.54) at a frequency of 10 Hz and square wave frequency (**d**).

Effect of accumulation potential on the oxidation peak current of FA was examined at different deposition potentials in the potential range of −0.60 to 0.20 V at a frequency of 10 Hz, a deposition time of 60 s, and a pH of 2.54. An increase of the oxidation peak current is observed on varying the accumulation potential from −0.60 V to a maximum value at −0.40 V. On further increase of the electrode potential to 0.20 V, a decrease in peak current is obtained. Therefore, the accumulation potential of −0.40 V is selected as the optimal value for subsequent experiments (Figure 4a).

#### 2.2.2. Effect of Accumulation Time

To evaluate the effect of accumulation time for the determination of FA, SWVs at PPy-MWCNTs/GCE at different accumulation times are performed. SWVs of FA were recorded at different times from 30 s to 180 s at a frequency of 10 Hz, deposition potential of −0.4 V, and pH of 2.54. The oxidation peak current increases on increasing time from 30 s to 60 s, then a decrease of the peak current is observed on any further increase of time. This suggests that the amount of adsorbed FA on the modified electrode surface reaches a maximum value at 60 s (Figure 4b). Therefore, the accumulation time of 60 s is selected for further studies.

#### 2.2.3. Effect of pH

To study the effect of pH on the determination of FA, SWVs at PPy-MWCNTs/GCE at different pH values were carried out at a frequency of 10 Hz, deposition potential of −0.40 V, and deposition time of 60 s. The behavior was examined at a pH range of 2.54 to 5.51. At a pH of 2.54, the peak current amounts, *i*_p_, to 4.28 µA. On increasing the pH of the solution to 3.51, *i*_p_ increases slightly and on further increase, it decreases sharply (Figure 4c). Thus, pH 3.5 is chosen in our work.

#### 2.2.4. Effect of Square Wave Frequency

In order to decrease the determination limit of FA using the proposed method, SWVs were performed at a deposition potential of −0.40 V, pH of 3.5, and deposition time of 60 s, and the frequencywas changed from 5 Hz to 40 Hz (Figure 4d). A linear relationship was obtained between the frequency and peak current, and a frequency of 30 Hz was chosen to be the measurement frequency.

### 2.3. Calibration Curve and Detection Limit

**Figure 5:**
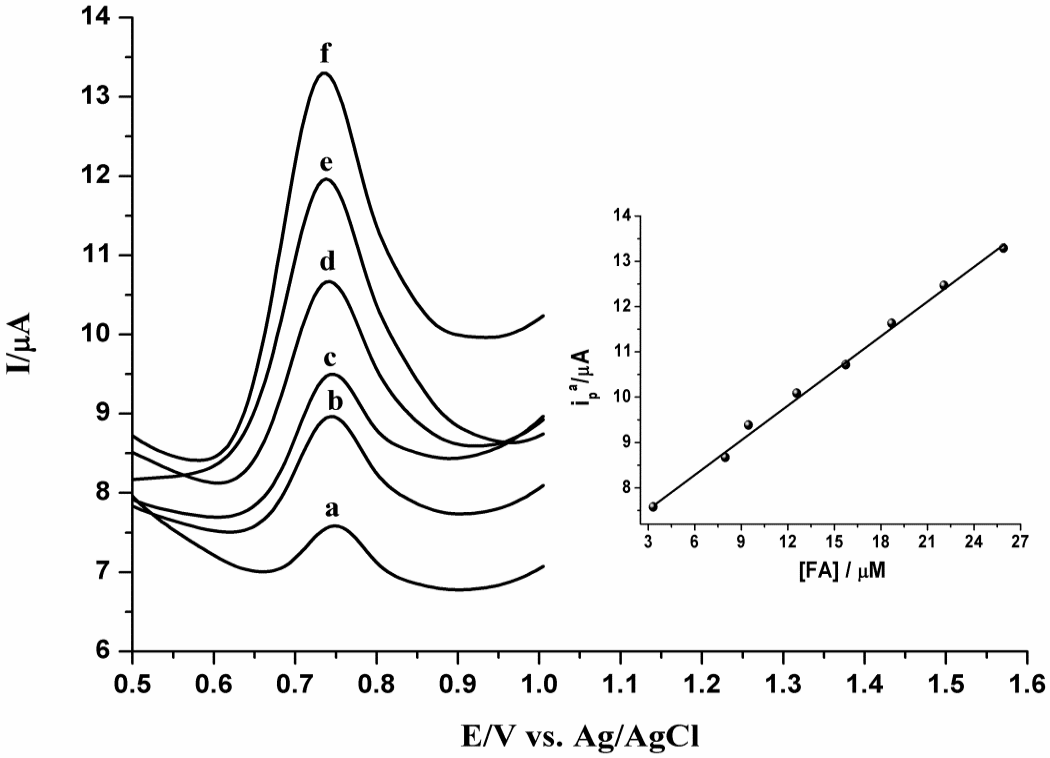
SW voltammograms obtained under optimal conditions of pH 3.5, accumulation potential of −0.40 V, accumulation time of 60 s, and frequency of 30 Hz in 0.2 M H_3_PO_4_ solution containing different concentrations of FA (concentration increases from curve a to curve f: 3.32, 7.96, 9.46, 15.70, 18.70 and 25.90 × 10^−6^ M, respectively). Inset: Calibration curve for FA on a PPy-MWCNTs/GC-modified electrode.

The square wave voltammograms obtained at PPy-MWCNTs/GCE in 0.2 M H_3_PO_4_ solution at optimal conditions (pH 3.5, accumulation potential −0.40 V, accumulation time 60 s and frequency 30 Hz) on successive additions of ferulic acid were recorded. Figure 5 shows the typical SWVs for FA at different concentrations. The oxidation peak current, *i*_p_, of ferulic acid increases linearly with an increasing concentration from 3.32 × 10^−6^ to 2.59 ×10^−5^ M. This is represented by the following linear regression Equation (2):

(2) *i*_p_ (*A*) = 6.79 × 10^−^^6^ + 0.255 *C*_FA_ (µM), *r* = 0.996

From the analytical data, the lower limit detection value for FA is determined to be 1.17 × 10^−6^ M (*S/N* = 3). This is ascribed not only to the large surface area of the PPy-MWCNTs/GCE, but also to the adsorption of FA, as well as the electro-catalytic effect of the modified electrode.

### 2.4. Interferences

The influence of interference was evaluated. Ascorbic acid was added into the cell containing 7.96 × 10^−6^ M FA. Experimental results showed that a 100-fold concentration of ascorbic acid did not interfere with the determination of ferulic acid (signal change below 5%). The results indicated that the present constructed sensor of PPy-MWCNTs/GCE was adequate for the determination of ferulic acid.

### 2.5. Analytical Application

**Figure 6:**
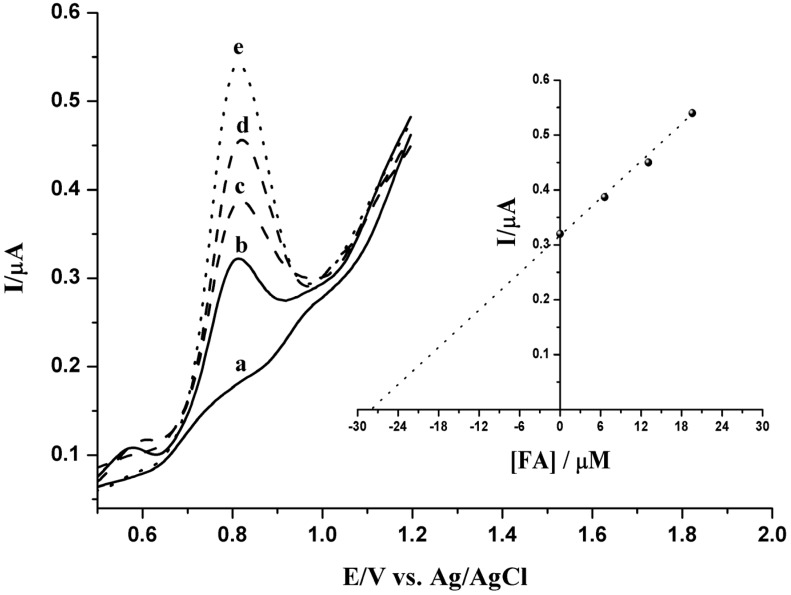
Square wave voltammogramic response of commercial popcorn in 0.2 M H_3_PO_4_ solution at optimal conditions: pH 3.5, accumulation potential −0.40 V, accumulation time 60 s, and frequency 30 Hz: (**a**) background; (**b**) 50.0 μL of popcorn sample; (**c**,**d** and **e**) Successive additions of standard ferulic acid solution (6.62×10^−6^ M, 1.31×10^−5^ M and 1.96 × 10^−5^ M, respectively). Inset: Standard addition plot obtained for determination of FA in a commercial popcorn sample.

In order to evaluate the validity of the sensor based on Ppy-MWCNTs/GCE for the determination of ferulic acid in a real sample, the total content of FA in commercial popcorn is determined with the proposed method using the adsorptive square wave voltammetric technique. The total content of FA in the commercial popcorn sample was determined by using the standard addition method with a standard solution of FA, under the same procedure described earlier at the optimal parameters. Successive amounts of standard FA solution were added to the test solution and then the voltammograms are recorded. Typical results are depicted in Figure 6, where the solid SWV represents the blank signal and the real sample. The total content of ferulic acid of the dried commercial popcorn sample is expressed as milligrams of ferulic acid equivalents (FAE) per g. It is estimated to be 3.26 mg/g.

## 3. Experimental Section

### 3.1. Treatment of MWCNTs

MWCNTs were sonicated with a mixture of H_2_SO_4_ and HNO_3_ (3/1, volume ratio) for 6 h to remove impurities, reduce bundle sizes, and to generate functional groups on their surface. The treated MWCNTs were then washed several times with bi-distilled water until the washing was neutral and then dried at about 70 °C. Mixture of 5 mg treated MWCNTs, 30 mg sodium dodecyl sulfate, 1 mL *N*,*N*-dimethylformamide, and 1 mL ethanol was ultrasonically mixed for 4 h to form a stable black suspension.

### 3.2. Preparation of PPy-MWCNTs/GCE

**Figure 7:**
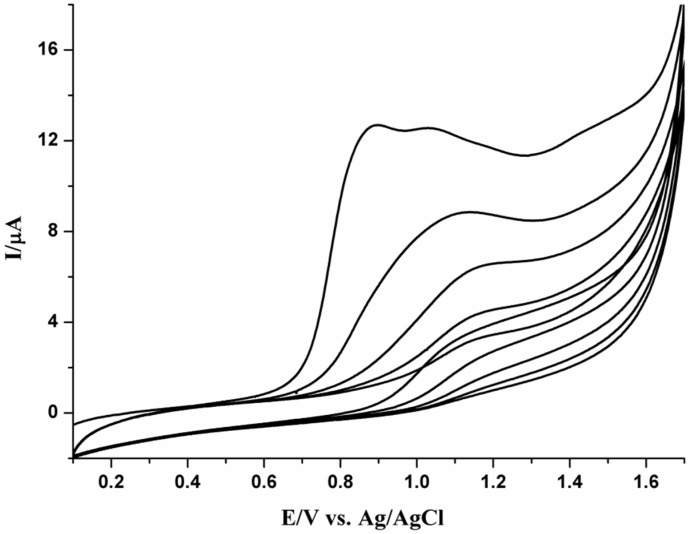
Cyclic voltammogram of 1.0 × 10^−4^ M pyrrole and 50 µL MWCNTs on GCE in 0.2 M H_3_PO_4_ (pH 2.54) for five cycles at scan rate of 20 mV s^−1^.

The polypyrrole-multiwalled carbon nanotubes/glassy carbon electrode (PPy-MWCNTs/GCE) was prepared and used as the working electrode. Platinum wire was used as counter electrode. A silver/silver chloride (KCl, 1 M) electrode was employed as the reference electrode. Polypyrrole film doped with CNTs on glassy carbon electrode PPy-MWCNTs/GCE was fabricated by adding a certain amount of the treated MWCNTs suspension (50 µL) to a 1.0 × 10^−4^ M distilled pyrrole solution in 0.2 M H_3_PO_4_ (pH 2.54) under ultrasonic stirring. The reaction mixture was electrolyzed using two different techniques for comparison. First, an anodic potential of 0.95 V *versus* Ag/AgCl was applied for 30 s as described by Peng *et al.* [12]. Second, cyclic voltammetric polymerization in a potential range of 0.1 to 1.7 V for five cycles at scan rate of 20 mV s^−1^ was employed as illustrated in Figure 7. The details of electroplymerization by cyclic voltammetry have been described elsewhere [25,26,27]. The solutions were degassed by bubbling dry N_2_ gas for 10 min prior to polymerization and a N_2_ atmosphere maintained during the experiments. The prepared PPy-MWCNTs/GC electrode was washed repeatedly with bi-distilled water and methanol to remove the electrolyte and the monomer. The prepared PPy-MWCNTs/GCE was then transferred into 0.2 M H_3_PO_4_ solution blank and subsequent voltammetric cycling for five cycles was conducted for cleaning at room temperature. For comparison, the electrochemical activity of FA was tested on the two different fabricated electrodes by potentiostatic and potentiodynamic polymerizations, the voltammograms of FA were identical on both. Potentiodynamic technique has been utilized for the electropolymerization of PPy-MWCNTs-modified electrode.

### 3.3. Instruments

The electrochemical experiments were performed using an Autolab PGSTAT128N Potentiostat/Galvanostat (Eco Chemie BV, Utrecht, The Netherlands). Electrochemical analyzer was operated via NOVA 1.10 software (Metrohm Autolab B.V., Utrecht, The Netherlands). NOVA is a software package designed to control Autolab instruments with USB interface. Electrochemical measurements were performed using bare or modified glassy carbon electrode as working electrode. Glassy carbon electrode was freshly polished to a smooth surface using fine grades of SiC paper (0.5 µm) and washed with bi-distilled water. After sonication cleaning in water for 2 min, the electrode was modified. Between measurements, the electrode surface was polished and sonicated to get a clean surface and to avert any possible problems from the adsorption of FA oxidation reaction products onto the electrode surface. Thus, a renewal working electrode was done before each measurement. Surface morphologies examination of polypyrrole and polypyrrole-MWCNTs composite films was performed using scanning electron microscope, JOEL (JSM T200, Tokyo, Japan) with electron beam energy of 15 kV. For this purpose, deposition of thin layer of gold (50 Ǻ) was carried out using physical vapour deposition. pH measurements were performed using a HI 2210 benchtop pH-meter (HANNA Instruments, Bucharest, Romania) with a combined pH reference electrode.

### 3.4. Sample Treatment

Accurate weight of dried powder of popcorn sample (4.0 g) was transferred into a 50 mL conical flask, and then ethanol was added and sonicated for 2 h at 50 °C. The mixture was cooled to room temperature and filtered off through a Whatman paper (No. 1). Dilution of the filtrate to 8 mL with ethanol was done. Then 50.0 μL of aliquot sample solution was added directly to 15.0 mL of H_3_PO_4_ in the electrochemical cell and the square wave voltammogram was recorded.

## 4. Conclusions

The glassy carbon electrode-modified with polypyrrole-multiwalled carbon nanotubes (PPy-MWCNTs/GCE) was prepared and used as a working sensor for the detection of ferulic acid. Cyclic and square wave voltammetric techniques were used to investigate the electrochemical behavior of ferulic acid. It was shown that PPy-MWCNTs/GCE exhibits remarkable electrochemical effects towards the oxidation of FA. The modified electrode, PPy-MWCNTs/GCE, enhances the oxidation peak current of FA. The results can be applied for the adsorptive stripping voltammetric determination of FA concentrations in a commercial sample of popcorn.
